# Supplementary material for: Molecular Classification to Prognosticate Response in Medically Managed Endometrial Cancers and Endometrial Intraepithelial Neoplasia
Source: Cancers (Basel). 2021 Jun 7;13(11):2847. doi: 10.3390/cancers13112847 (PMC8201008; doi:10.3390/cancers13112847)
Supplement: Supplementary file 1 [file cancers-13-02847-s001.zip › cancers-1179673-supplementary.pdf]

cancers

Supplementary Materials: Molecular Classification to Prognosticate Response in Medically Managed Endometrial Cancers and Endometrial Intraepithelial Neoplasia

Allison M. Puechl, Daniel Spinosa, Andrew Berchuck, Angeles Alvarez Secord, Kerry E. Drury, Gloria Broadwater, Janice Wong, Regina Whitaker, Nicolas Devos, David L. Corcoran, Kyle C. Strickland and Rebecca A. Previs

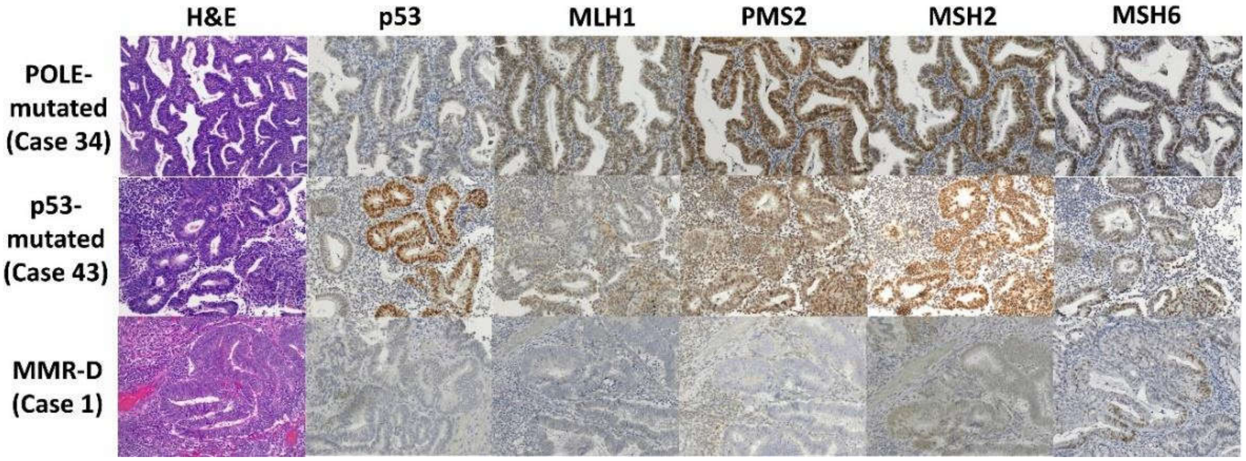

Figure S1. Representative H&E, p53, and mismatch repair protein (MLH1, PMS2, MSH2, and MSH6) staining of a case with a *POLE* mutation (case 34), *TP53* mutation (case 43), and mismatch repair deficiency (case 1). Case 34 demonstrates an example of a case with wild-type staining for p53 and intact protein expression for the mismatch repair proteins. Case 43 represents a tumor with an underlying pathogenic p53 mutation, which had strong and diffuse p53 staining and intact expression of mismatch repair proteins. Case 1 was consistent with a mismatch repair deficiency, showing loss of MLH1 and PMS2 expression but retained focal expression of MSH2 and MSH6. Photomicrographs (H&E and IHC) were obtained at 20× magnification. Abbreviations: MMR-D, mismatch repair deficient.

Table S1. Clinical and pathologic follow-up of patients treated with LNG-IUS for endometrial cancer or endometrial intraepithelial neoplasia. Abbreviations: MMR, mismatch repair; PR, partial response; SD, stable disease.

	Molecular Classification				
	**MMR-deficient (*n* = 6)**	***POLE*-mutated (*n* = 4)**	**p53-abnormal (*n* = 4)**	**p53 wild type (*n* = 44)**	**Total (*n* = 58)**
**Number of biopsies per patient**					
Median (range)	2 (0–3)	1 (1–3)	1 (1–2)	1 (0–4)	1 (0–4)
**Pathologic response from subsequent endometrial biopsies (*n*)**					
Benign complete response	3	5	2	33	43
Atypical (hormonal effect); PR	1	-	-	17	18
Atypical (improved disease); PR	1	-	1	5	7
Atypical (no better/no worse); SD	3	-	1	11	15
Atypical progressive disease	-	1	1	6	8
Insufficient tissue to evaluate	3	-	-	3	6
Total number of biopsies	11	6	5	75	97
**Time to first biopsy (months)**					
Median (range)	3.4 (3.1 – 6.0)	14.1 (3.4 – 73.7)	3.3 (3.0 – 6.0)	4.6 (2.2 – 25.0)	4.4 (2.2 – 73.7)
**Time to progression or definitive therapy (months)**					
Median (range)	20.9 (7.5–34.4)	21.4 (21.4–21.4)	5.7 (4.6–6.8)	7.8 (2.5–40.0)	7.5 (2.5–40.0)

**Duration of follow-up (months)**					
Median (range)	18.0 (15.2–65.8)	38.9 (20.6–106.5)	9.2 (4.0–53.0)	34.3 (3.3–141.5)	28.8 (3.3–141.5)
